# Effect of Storage Temperature on Key Functions of Cultured Retinal Pigment Epithelial Cells

**DOI:** 10.1155/2015/263756

**Published:** 2015-09-13

**Authors:** Lara Pasovic, Jon Roger Eidet, Berit S. Brusletto, Torstein Lyberg, Tor P. Utheim

**Affiliations:** ^1^Department of Medical Biochemistry, Oslo University Hospital, Kirkeveien 166, P.O. Box 4956, Nydalen, 0424 Oslo, Norway; ^2^Faculty of Medicine, University of Oslo, Sognsvannsveien 9, 0372 Oslo, Norway; ^3^Department of Oral Biology, Faculty of Dentistry, University of Oslo, Sognsvannsveien 10, P.O. Box 1052, Blindern, 0316 Oslo, Norway

## Abstract

*Purpose*. Replacement of the diseased retinal pigment epithelium (RPE) with cells capable of performing the specialized functions of the RPE is the aim of cell replacement therapy for treatment of macular degenerative diseases. A storage method for RPE is likely to become a prerequisite for the establishment of such treatment. Herein, we analyze the effect of storage temperature on key functions of cultured RPE cells. *Methods*. Cultured ARPE-19 cells were stored in Minimum Essential Medium at 4°C, 16°C, and 37°C for seven days. Total RNA was isolated and the gene expression profile was determined using DNA microarrays. Comparison of the microarray expression values with qRT-PCR analysis of selected genes validated the results. *Results*. Expression levels of several key genes involved in phagocytosis, pigment synthesis, the visual cycle, adherens, and tight junctions, and glucose and ion transport were maintained close to control levels in cultures stored at 4°C and 16°C. Cultures stored at 37°C displayed regulational changes in a larger subset of genes related to phagocytosis, adherens, and tight junctions. *Conclusion*. RPE cultures stored at 4°C and 16°C for one week are capable of maintaining the expression levels of genes important for key RPE functions close to control levels.

## 1. Introduction

The retinal pigment epithelium (RPE) is a highly specialized tissue. Situated between the neuroretina and choroid, it performs several functions that are crucial for supporting sight. Among the most important are phagocytosis of shed photoreceptor (PR) outer segments, regeneration of the visual cycle pigment rhodopsin, transportation of glucose and nutrients from the choroid to the distal part of the neuroretina, and transportation of excess fluid in the opposite direction [1, 2]. Malfunction of the RPE, implying a disrupted ability to perform these tasks, is a direct cause of prevalent retinal diseases like age-related macular degeneration (AMD) [3, 4] and a consequence of inherited disorders like Stargardt disease [5].

A promising approach for treatment of these diseases is the transplantation of tissue engineered RPE [6–10]. However, for the prospect of tissue engineering to become a widespread treatment option, it is necessary to ensure cell availability during short-term storage and transportation of RPE cells. In the process of establishing such a protocol, our research group has demonstrated that storage temperature has a crucial impact on the viability and morphology of cultured RPE cells [11]. ARPE-19 cultures stored at 16°C displayed the greatest number of viable cells compared to cells stored at eight other temperatures (4°C, 8°C, 12°C, 20°C, 24°C, 28°C, 32°C, and 37°C) after seven days of storage [11].

Having established the potential effect of storage temperature on cell viability, we herein aim to investigate the effect of storage temperature on the gene expression associated with many highly specialized functions of the RPE, using microarray technology. Increased knowledge of the effects of storage on cultured ARPE-19 cells is imperative for future use of RPE transplantation in treatment of eye diseases affecting millions of people worldwide [12].

## 2. Materials and Methods

### 2.1. Cell Culture Media and Reagents

Adult retinal pigment epithelial cells (ARPE-19) were purchased from the American Type Culture Collection (ATCC) (Manassas, VA). Dulbecco's Modified Eagle's Medium (DMEM), Nutrient Mixture F12, fetal bovine serum (FBS), trypsin-EDTA, phosphate-buffered saline (PBS), 4-(2-hydroxyethyl)-1-piperazineethanesulfonic acid (HEPES), sodium bicarbonate, gentamycin, penicillin, and streptomycin were from Sigma-Aldrich (St. Louis, MO). Minimum Essential Medium (MEM) was purchased from Invitrogen (Carlsbad, CA). Nunclon T25 and T75 flasks, pipettes, and other routine plastics were purchased from VWR (West Chester, PA). The miRNeasy Mini Kit containing the QIAzol Lysis Reagent was obtained from Qiagen (Venlo, Netherlands).

### 2.2. Cell Culture and Storage

RPE cells from the ARPE-19 cell line were cultured under standard conditions in 95% air and 5% CO_2_ at 37°C in DMEM/F12 medium containing 10% FBS, 50 units/mL penicillin, and 50 *μ*g/mL streptomycin. All ARPE-19 cells were from passage 4 and lower after acquisition from the vendor. Upon reaching confluence, the cells were seeded (5000 cells/cm^2^) in Nunclon T25 and T75 flasks. The culture medium was changed after two days, and confluent cultures were obtained on the third day. Three cultures were immediately processed for mRNA amplification and used as controls, while nine cultures were prepared for storage. The cells were rinsed with PBS, and the culture medium was replaced by storage medium consisting of MEM, 25 mM HEPES, 22.3 mM sodium bicarbonate, and 50 *μ*g/mL gentamycin, hereafter referred to as MEM. The cultures were then placed in storage containers maintaining a stable temperature of either 4°C, 16°C, or 37°C and stored for seven days. The configuration and design of the custom-made storage containers have been explained earlier [11].

### 2.3. RNA Extraction and Microarray Hybridization

Cultured ARPE-19 cells that had been stored for seven days at 4°C, 16°C, and 37°C, as well as control cultures that had not been stored, were rinsed with PBS and directly lysed with QIAzol Lysis Reagent. 150 ng of total RNA was subjected to GeneChip HT One-Cycle cDNA Synthesis Kit and GeneChip HT IVT Labeling Kit, following the manufacturer's protocol for whole genome gene expression analysis (Affymetrix, Santa Clara, CA, USA). Microarray analyses were performed using the Affymetrix GeneChip Human Gene 1.0 ST Arrays (Affymetrix, Santa Clara, CA), which contains approximately 28,000 gene transcripts. Biotinylated and fragmented single stranded cDNAs were hybridized to the GeneChips. The arrays were washed and stained using FS-450 fluidics station (Affymetrix). Signal intensities were detected by Hewlett Packard Gene Array Scanner 3000 7G (Hewlett Packard, Palo Alto, CA, USA).

The scanned images were processed using the AGCC (Affymetrix GeneChip Command Console) software and the CEL files were imported into Partek Genomics Suite software (Partek, Inc. MO, USA). The Robust Multichip Analysis (RMA) algorithm was applied for generation of signal values and normalization. Gene transcripts with maximal signal values of less than 32 across all arrays were removed to filter for low and nonexpressed genes, reducing the number of gene transcripts to 17,684. For expression comparisons of different groups, profiles were compared using a 1-way ANOVA model. The results were expressed as fold changes (FC) and *P* values.

### 2.4. Microarray Data Analysis

Gene networks and canonical pathways representing key genes were identified using Ingenuity Pathways Analysis (IPA) (http://www.ingenuity.com/). Briefly, the data set containing gene identifiers and corresponding fold changes and *P* values was uploaded into the web-delivered application and each gene identifier was mapped into its corresponding gene object in the Ingenuity Pathways Knowledge Base (IPKB). The functional analysis identified the biological functions and/or diseases that were most significant to the data sets. Fisher's exact test was performed to calculate a *P* value determining the probability that each biological function and/or disease assigned to the data set was due to chance alone. The data sets were mined for significant pathways with the IPA library of canonical pathways and networks were generated by using IPA as graphical representations of the molecular relationships between genes and gene products.

The presentation of the microarray data was divided into two manuscripts: the present and another addressing the genes not presented herein. This was done to allow for a more profound discussion of our findings.

### 2.5. Validation by PCR

The differential gene expression data were validated for selected transcripts (TYRP1, DSC1, and GLUT12) using TaqMan Gene Expression Assays and the Applied Biosystems ViiA 7 real-time PCR system (Applied Biosystems, Life Technologies). Briefly, 200 ng of total RNA was reverse transcribed using qScript cDNA Super Mix (Quanta Biosciences) following the manufacturer's instructions. After completion of cDNA synthesis, 1/10th of the first strand reaction was used for PCR amplification. A total of 9 *μ*L of diluted cDNA (diluted in H_2_O), 1 *μ*L of selected primer/probes TaqMan Gene Expression Assays (Life Technologies), and 10 *μ*L TaqMan Universal Master Mix (Life Technologies) were used following the manufacturer's instructions. Transducin-like enhancer of split 1 (TLE1) was used as endogenous control due to low coefficient of variation (CV) (0.444) in the Affymetrix study. Each gene was run in duplicates. TaqMan Gene Expression Assays (Life Technology) used assays detecting TYRP1 (Hs00167051_m1), DSC1 (Hs00245189_m1), GLUT12 (Hs01547015_m1), and TLE1 (Hs00270768_m1).

*P* values were calculated using Student's *t*-test in Microsoft Excel using delta Ct values. Normalized target gene expression levels (FC) were calculated using the formula: 2^−ΔΔCt^.

## 3. Results

### 3.1. Analysis of Retinal Pigment Epithelial Cell Functions

**Figure 1:**
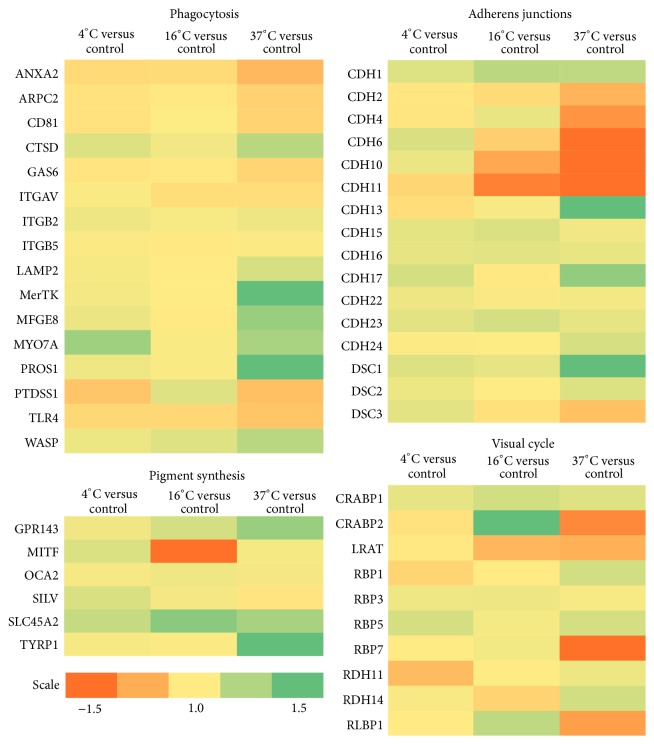
Heat map diagrams of a selection of the most important genes related to RPE phagocytosis, pigment synthesis, adherens junctions, and visual cycle, respectively. The color scale illustrates the relative expression level of mRNAs: green color represents a high expression level and orange color represents a low expression level.

**Figure 2:**
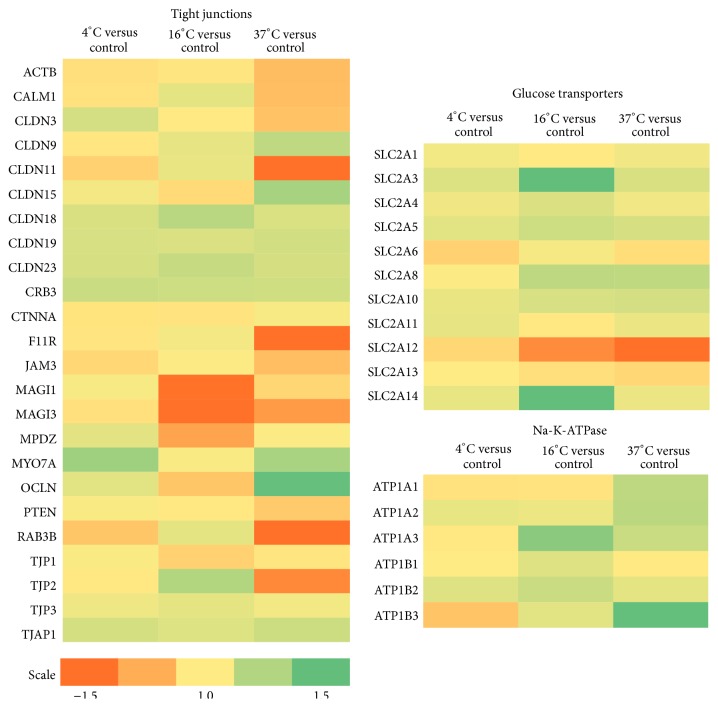
Heat map diagrams of a selection of the most important genes related to RPE tight junctions, glucose transportation, and Na-K-ATPase, respectively. The color scale illustrates the relative expression level of mRNAs: green color represents a high expression level and orange color represents a low expression level.

In order to elucidate the expression patterns of genes critical to important RPE functions, we investigated the expression levels of individual genes associated with distinctive cellular properties (i.e., phagocytosis, pigment synthesis, visual cycle, adherens and tight junctions, and glucose and ion transport). Only significantly regulated genes are mentioned, namely, those displaying a *P* value below 0.05. Results are presented in Table 1 and Figures 1-2.

*Phagocytosis*. Phagocytosis of photoreceptor (PR) outer segments is a crucial function of the RPE, and the components of its phagocytic machinery have been thoroughly described [13]. Compared to control cells, cells that had been stored at 4°C and 16°C showed no difference in levels of expression for any of the 16 identified genes important for phagocytic functions. Cells that had been stored at 37°C displayed significant changes in gene regulation of several genes; however, only the engulfment-related gene PROS1 displayed a fold change of more than 1.5. 

*Pigment Synthesis*. Production of melanin pigment by the RPE has two important functions in vivo: photoprotection, due to the antioxidant effect of melanin, and prevention of internal reflection of light from the sclera back to the retina [14]. Of six identified genes related to pigment synthesis, SLC45A2 showed a 1.4-fold increase and MITF a 2.3-fold decrease in expression at 16°C compared to controls. TYRP1 showed a notable 3.6-fold upregulation at 37°C compared to control cells. At 4°C there were no significant differences in expression levels. 

*Visual Cycle*. The RPE serves a crucial function in the visual cycle by reisomerizing all-trans-retinal to 11-cis-retinal, and defects in key proteins of the cycle can in themselves lead to various retinal diseases [1, 15]. Most of the identified visual cycle genes were maintained at expression levels similar to controls. RLBP1 and RBP7 expression was decreased 1.3-fold and 1.6-fold, respectively, in cells stored at 37°C, while RDH11 was decreased 1.2-fold in cells stored at 4°C. In cultures stored at 16°C, CRABP2 (RBP6) expression was increased 1.8-fold. 

*Adherens Junctions*. Adherens junctions link actin filaments between epithelial cells and provide a strong mechanical attachment in cellular monolayers. Cadherins form homodimers with cadherins of adjacent cells and are pivotal for the integrity of the junction [16]. A total of 13 different cadherins were identified in our data set, and their expression levels were unchanged in cells stored at 4°C and 16°C compared to the control. At 37°C, cadherins 6, 10, and 11 were downregulated 3.0-fold, 1.7-fold, and 2.2-fold, respectively, while DSC1 and CDH13 were upregulated 8.3-fold and 2.0-fold, respectively, compared to control cells. 

*Tight Junctions*. Tight junctions of the RPE regulate cell polarity, proliferation, and paracellular diffusion, and they are constituents of the blood-retinal barrier [17]. Of the 23 identified genes involved in the tight junction complex, seven were differentially expressed in cells stored at 16°C, but only the downregulation of MAGI1 and MAGI3 exceeded a fold change of 1.5. A total of 14 genes were differentially expressed at 37°C, of which CLDN11, F11R, and RAB3B were downregulated more than 1.5-fold. CRB3 was increased 1.2-fold in cells stored at 4°C.

*Glucose Transport*. The RPE is critical for supplying the inner part of the retina with glucose, and the maintenance and regulation of GLUT channels are essential for this function [18, 19]. In cells that had been stored at 16°C, we found an increased expression of three of a total of 11 glucose transporter isoforms identified in our material. GLUT3 was increased 2-fold, GLUT8 1.2-fold, and GLUT14 1.6-fold. In cells stored at 37°C, there was a 1.2-fold increase in expression of GLUT8 and a 2.7-fold decrease of GLUT12. No changes in expression were detected at 4°C.

*Ion Transport*. The Na-K-ATPase establishes and maintains electrochemical gradients across the plasma membrane [20], thereby providing the energy for transepithelial transport [15]. Of six identified genes involved in the Na-K-ATPase, ATP1A3 and ATP1B2 were upregulated 1.4-fold and 1.2-fold at 16°C storage, respectively. ATP1A1 and ATP1B3 were upregulated 1.2-fold and 1.5-fold, respectively, at 37°C storage. There were no significant changes at 4°C compared to controls.

### 3.2. PCR Validation of Key Genes

**Figure 3:**
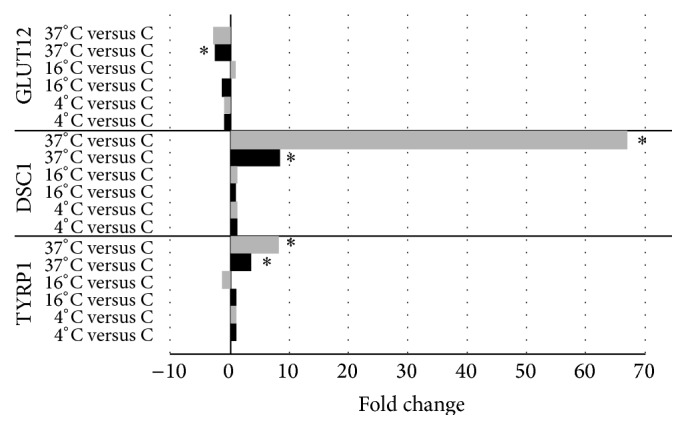
Validation of microarray expression results by qRT-PCR. Selected mRNAs (TYRP1, DSC1, and GLUT12) were differentially expressed in cultured RPE cells stored at different temperatures (4°C, 16°C, or 37°C) compared to control cells that had not been stored. Black bars indicate microarray expression values and grey bars represent PCR verification values. ^*∗*^ *P* < 0.01.

Relative quantification of a few key genes (TYRP1, DSC1, and GLUT12) was performed with real-time PCR (Figure 3). The expression of TYRP1 was significantly upregulated to 8.2-fold at 37°C compared to controls. In comparison, the microarray data showed a 3.6-fold upregulation of this gene at 37°C. DSC1 expression was significantly and considerably upregulated in the 37°C group compared to controls, with a 67.0-fold upregulation. This is higher than the corresponding microarray data, which yielded an 8.3-fold upregulation at this temperature. PCR analysis of GLUT12 expression showed a similar downregulation compared to microarray results (3.0-fold and 2.7-fold, resp.). However, results were nonsignificant in the PCR group (*P* value = 0.068). PCR validation showed that expression of TYRP1, DSC1, and GLUT12 was not significantly regulated in the 4°C and 16°C culture groups, which is in line with the microarray data.

## 4. Discussion

In this study, we investigated the effect of storage temperature on important cellular functions of ARPE-19 cells by comparing the expression levels of genes associated with phagocytosis, pigment synthesis, visual cycle, adherens and tight junctions, and glucose and ion transport.

The ARPE-19 cell line is recognized for displaying significant functional differentiation and forming polarized epithelial monolayers and tight junctions with barrier properties [21, 22]. However, the cell line does not mirror all the functions and characteristics of native RPE [23–25]. Some studies have demonstrated a relatively lower expression of some RPE-specific transcripts in ARPE-19 cells compared to native RPE cells [26], while others have not [27]. Native RPE exhibits considerable regional variation, and thus any culture models will be inherently heterogeneous [23, 28, 29]. Cells and cell lines in culture can exceed the normal variation described in RPE in vivo [23, 30–32]. Gene expression by cultured RPE cells is substrate dependent [33], and ARPE-19 grown on plastic displays the phenotype closest to native RPE, capable of yielding a functional profile of differentially expressed genes [34]. The global expression profile of ARPE-19 cells can also be directed towards that of primary RPE cells by withdrawing serum [24]. In the present study, cells were cultured and stored on plastic, and the storage medium contained no xenobiotic components.

Phagocytosis of shed photoreceptor outer segments is vital to photoreceptor repair and represents one of the most critical functions of the RPE [1, 35]. We found no changes in expression of phagocytosis-associated genes after storage at 4°C and 16°C compared to control cells (Table 1). Two receptor ligand pairs are recognized for exhibiting key roles in the molecular machinery of RPE phagocytosis. These include the receptor tyrosine kinase MerTK and its secreted ligands Gas6 and Protein S, as well as the integrin receptor *α*V*β*5 and its secreted ligand MFG-E8 [36]. ARPE-19 cells are capable of phagocytosing photoreceptor outer segments [37–39], but some differences exist compared to primary cultures. Both require the integrin receptor *α*v*β*5 for the binding and internalization of outer segments [21, 37], the main difference being observed at the level of promoter strength, yielding much higher transcriptional activity in ARPE-19 [40]. With the exception of Protein S, expression of all of these important genes was maintained during storage at all temperatures. Although the differences in expression of the remaining phagocytosis associated genes were modest, these results may indicate a slightly disrupted phagocytic ability in cells stored at 37°C.

The expression of genes associated with pigment synthesis in the RPE was also evaluated due to its many functions, including protection from oxidative stress [41–43]. Four genes have been described as key contributors in the melanin biosynthesis pathway: TYR, TYRP1, TYRP2, and P gene (OCA2) [44]. Smith-Thomas et al. [14] found that primary human RPE cells failed to express TYRP2 and that a very low percentage of the cells expressed TYRP1, but only if cultured for more than 3 weeks. Lu et al. [44] found that human RPE cultured under standard conditions failed to express any of the four key genes mentioned above. However, we were able to detect both OCA2 and TYRP1 in all culture groups, as well as several other genes related to pigment synthesis (Table 1).

Upon transduction of light energy into electrical impulses in the PR, 11-cis-retinal is converted to all-trans-retinal, which is cycled to the RPE for reisomerization [15]. A string of proteins contributes in the visual cycle, and the expression levels of critically important proteins such as cellular retinol binding protein 1 (RBP1, also known as CRBP1), lecithin retinol acyltransferase (LRAT), cellular retinaldehyde binding protein 1 (RLBP1, also referred to as CRALBP), and cellular retinol binding protein 5 (RBP5) were maintained at control levels during storage at all three temperatures. This indicates that the visual cycle can be preserved under the storage conditions used in this study.

Cell-cell adhesion is important for maintaining the correct RPE phenotype [45, 46]. Cultures stored at 4°C and 16°C did not differ from controls in regard to expression of adherens junction genes. Cultures stored at 37°C, however, showed a differential regulation of five adherens junction genes, among them an 8.3-fold upregulation of DSC1 and a change in expression of several cadherins. These changes might indicate a slight perturbance of adherens junction properties after 37°C storage. This group also showed the largest expression changes of tight junction genes, mostly downregulation. This might indicate a loss of integrity of the intercellular junction in cells stored at 37°C compared to control cells. The classic tight junction proteins ZO-1 and occludin did not display any changes in expression levels after storage at any of the three temperatures.

In a previous study, we demonstrated that the number of viable ARPE-19 cells at 4°C storage dropped to less than 4% compared to the control group [11]. In the present study, we find few differences between the 4°C group and the control. This seemingly contradictory finding can have at least two explanations. First, the cultures stored at 4°C contain a large number of dead and dying cells, which have a tendency to detach and be washed away during preparations, thereby not being included in the analysis. Second, temperature has a crucial effect on the adhesive abilities of several cell types [47–50] and adhesion seems to be severely affected during 4°C storage, resulting in the loss of otherwise viable and well-functioning cells from the monolayer. Unpublished data from our research group demonstrates improved viability following storage at 4°C by implementing a radical change in the culture protocol in order to improve cell adhesion. This finding supports our hypothesis that cellular adhesion is severely affected at low storage temperatures.

We also assessed the expression of glucose and ion transporters. Several GLUT proteins were identified in our material, with GLUT1 expression being dominant (Table 1). This is in line with existing gene expression studies on native RPE [18, 51, 52]. Expression of GLUT1 was maintained at control levels during storage at all temperatures. Given the dominant role of this transporter in RPE cells, the maintenance of its expression in all storage groups indicates a preservation of glucose transport function after storage. In an earlier study by Takagi et al. [52], the addition of FBS to the culture medium was shown to increase the expression of GLUT1 in human RPE cells. Based on this observation, one might anticipate a downregulation of this isoform when replacing the FBS-containing growth medium with a xenobiotic-free storage medium. However, that was not the case in our cultures. Expression of GLUT3 was increased 2-fold after storage at 16°C. GLUT3 is highly effective, displaying both a higher affinity and a fivefold greater transport capacity for glucose than other isoforms including GLUT1 [53]. Its expression has been identified in several cell types characterized by very specific and high metabolic demand, such as neurons and placental trophoblasts [53–55]. Its expression in neurons increases in an activity-related manner to meet an increased demand [53]. We speculate whether this strategy is utilized by ARPE-19 cells stored at 16°C and if it contributes to preserving a larger number of viable cells compared to other temperatures where GLUT3 expression remains unchanged.

Active transport of Na^+^ across the apical membrane of RPE cells creates a high Na^+^ concentration in the subretinal space, which is crucial for the photoreceptor dark current and for transport of solutes through symporters and antiporters of the RPE [17]. Three isoforms of each of the Na-K-ATPase *α* and *β* subunits were identified, and most were expressed close to control levels in all storage groups. The same isoforms were identified in a recent study on native RPE [51].

## 5. Conclusion

When comparing the expression levels of genes involved in important RPE functions, it is evident that cells stored at 37°C display expression changes in a larger number of genes than cells stored at 4°C and 16°C. In conclusion, the findings of this study show that cells stored at 4°C and 16°C are capable of maintaining expression levels of genes important for key RPE functions close to the control levels.

## Figures and Tables

**Table 1:** Expression of genes involved in key functions of the RPE at different temperatures compared to controls.

Gene symbol	Gene description	Fold change		
		4°C versus C	16°C versus C	37°C versus C
Phagocytosis				
ANXA2	Annexin A2	−1.07	−1.06	−1.21
ARPC2	Actin related protein 2/3 complex, subunit 2, 34 kDa	−1.04	−1.01	**−1.10**
CD81	CD81 molecule	−1.04	1.01	−1.10
CTSD	Cathepsin D	1.11	1.05	** 1.23**
GAS6	Growth arrest-specific 6	−1.03	−1.01	−1.09
ITGAV	Integrin, alpha V	1.02	−1.05	−1.05
ITGB2	Integrin, beta 2	1.06	1.03	1.06
ITGB5	Integrin, beta 5	1.01	−1.01	1.01
LAMP2	Lysosomal-associated membrane protein 2	1.03	−1.00	**1.13**
MerTK	MER protooncogene, tyrosine kinase	1.04	1.00	1.73
MFGE8	Milk fat globule-EGF factor 8 protein	1.07	−1.01	1.32
MYO7A	Myosin VIIa	1.31	1.02	1.28
PROS1	Protein S	1.06	1.02	**1.73**
PTDSS1	Phosphatidylserine synthase 1	**−1.15**	1.11	**−1.18**
TLR4	Toll-like receptor 4	−1.07	−1.08	−1.15
WASP	Wiskott-Aldrich syndrome	1.06	1.10	**1.23**

Pigment synthesis				
GPR143	G protein-coupled receptor 143	1.05	1.14	1.33
MITF	Microphthalmia-associated transcription factor	1.12	**−2.35**	1.03
OCA2	Oculocutaneous albinism II	1.03	1.05	1.03
PMEL	Premelanosome protein	1.12	1.03	−1.02
SLC45A2	Solute carrier family 45, member 2	1.18	**1.37**	1.28
TYRP1	Tyrosinase-related protein 1	1.03	1.02	**3.62**

Visual cycle				
CRABP1	Cellular retinoic acid binding protein 1	1.08	**1.14**	1.11
CRABP2	Cellular retinoic acid binding protein 2	−1.03	**1.77**	**−1.40**
LRAT	Lecithin retinol acyltransferase	−1.01	−1.21	−1.23
RBP1	Retinol binding protein 1, cellular	−1.09	−1.00	1.15
RBP3	Retinol binding protein 3, interstitial	1.05	1.06	1.03
RBP5	Retinol binding protein 5, cellular	1.13	1.03	1.14
RBP7	Retinol binding protein 7, cellular	1.01	1.04	**−1.60**
RDH11	Retinol dehydrogenase 11	**−1.19**	1.00	1.06
RDH14	Retinol dehydrogenase 14	1.03	**−1.09**	**1.15**
RLBP1	Retinaldehyde binding protein 1	−1.00	1.21	**−1.31**

Adherens junctions				
CDH1	Cadherin 1, type 1, E-cadherin (epithelial)	1.11	1.22	1.21
CDH2	Cadherin 2, type 1, N-cadherin (neuronal)	−1.02	−1.07	−1.23
CDH4	Cadherin 4, type 1, R-cadherin (retinal)	−1.02	1.07	−1.35
CDH6	Cadherin 6, type 2, K-cadherin (fetal kidney)	1.12	−1.12	**−3.04**
CDH10	Cadherin 10, type 2 (T2-cadherin)	1.06	−1.27	**−1.73**
CDH11	Cadherin 11, type 2, OB-cadherin (osteoblast)	−1.08	−1.43	**−2.16**
CDH13	Cadherin 13	−1.05	1.03	**2.02**
CDH15	Cadherin 15	1.08	1.12	1.05
CDH16	Cadherin 16, KSP-cadherin	1.08	1.09	1.07
CDH17	Cadherin 17, LI cadherin (liver-intestine)	1.14	−1.02	1.35
CDH22	Cadherin 22, type 2	1.06	1.02	1.04
CDH23	Cadherin-related 23	1.09	**1.14**	1.08
CDH24	Cadherin 24, type 2	1.01	1.01	1.13
DSC1	Desmocollin 1	1.11	1.08	**8.34**
DSC2	Desmocollin 2	1.06	1.01	1.11
DSC3	Desmocollin 3	1.09	−1.04	**−1.17**

Tight junctions				
ACTB	Actin, beta	−1.05	−1.02	**−1.18**
CALM1	Calmodulin 1 (phosphorylase kinase, delta)	−1.04	**1.09**	**−1.18**
CLDN3	Claudin 3	1.14	−1.01	**−1.16**
CLDN9	Claudin 9	−1.02	1.08	**1.21**
CLDN11	Claudin 11	−1.11	1.07	**−2.91**
CLDN15	Claudin 15	1.04	−1.07	**1.28**
CLDN18	Claudin 18	1.12	**1.22**	1.12
CLDN19	Claudin 19	1.13	1.12	1.15
CLDN23	Claudin 23	1.13	**1.18**	1.14
CRB3	Crumbs family member 3	**1.17**	**1.17**	**1.15**
CTNNA	Catenin (cadherin-associated protein), alpha 1	−1.03	−1.03	1.03
F11R	F11 receptor	−1.02	1.04	**−1.52**
JAM3	Junctional adhesion molecule 3	−1.08	1.01	**−1.18**
MAGI1	Membrane associated guanylate kinase, WW and PDZ domain containing 1	1.03	**−1.50**	−1.08
MAGI3	Membrane associated guanylate kinase, WW and PDZ domain containing 3	−1.04	**−1.82**	**−1.33**
MPDZ	Multiple PDZ domain protein	1.09	**−1.29**	1.01
MYO7A	Myosin VIIA	1.31	1.02	1.28
OCLN	Occludin	1.09	−1.15	1.49
PTEN	Phosphatase and tensin homolog	1.02	−1.01	**−1.14**
RAB3B	RAB3B, member RAS oncogene family	−1.15	1.08	**−2.19**
TJP1	Tight junction protein 1	1.02	−1.10	−1.02
TJP2	Tight junction protein 2	−1.01	1.25	**−1.40**
TJP3	Tight junction protein 3	1.06	1.08	1.04

Glucose transport				
SLC2A1	Solute carrier family 2 (facilitated glucose transporter), member 1	1.04	−1.01	1.05
SLC2A3	Solute carrier family 2 (facilitated glucose transporter), member 3	1.11	**2.00**	1.13
SLC2A4	Solute carrier family 2 (facilitated glucose transporter), member 4	1.05	1.12	1.05
SLC2A5	Solute carrier family 2 (facilitated glucose transporter), member 5	1.10	1.16	1.13
SLC2A6	Solute carrier family 2 (facilitated glucose transporter), member 6	−1.11	1.03	−1.06
SLC2A8	Solute carrier family 2 (facilitated glucose transporter), member 8	1.01	**1.21**	**1.21**
SLC2A10	Solute carrier family 2 (facilitated glucose transporter), member 10	1.07	1.13	1.14
SLC2A11	Solute carrier family 2 (facilitated glucose transporter), member 11	1.08	−1.01	1.07
SLC2A12	Solute carrier family 2 (facilitated glucose transporter), member 12	−1.08	−1.39	**−2.69**
SLC2A13	Solute carrier family 2 (facilitated glucose transporter), member 13	1.01	−1.05	−1.08
SLC2A14	Solute carrier family 2 (facilitated glucose transporter), member 14	1.08	**1.58**	1.07

Na-K-ATPase				
ATP1A1	ATPase, Na^+^/K^+^ transporting, alpha 1 polypeptide	−1.04	−1.03	**1.21**
ATP1A2	ATPase, Na^+^/K^+^ transporting, alpha 2 polypeptide	1.08	1.06	1.22
ATP1A3	ATPase, Na^+^/K^+^ transporting, alpha 3 polypeptide	−1.01	**1.38**	1.17
ATP1B1	ATPase, Na^+^/K^+^ transporting, beta 1 polypeptide	1.01	1.12	−1.01
ATP1B2	ATPase, Na^+^/K^+^ transporting, beta 2 polypeptide	1.10	**1.17**	1.09
ATP1B3	ATPase, Na^+^/K^+^ transporting, beta 3 polypeptide	−1.16	1.10	**1.49**

*P* values < 0.05 are marked in bold font.
